# Ecosystem Health Assessment in the Pearl River Estuary of China by Considering Ecosystem Coordination

**DOI:** 10.1371/journal.pone.0070547

**Published:** 2013-07-23

**Authors:** Xiaoyan Chen, Huiwang Gao, Xiaohong Yao, Zhenhua Chen, Hongda Fang, Shufeng Ye

**Affiliations:** 1 Key Laboratory of Marine Environment and Ecology (OceanUniversity of China), Ministry of Education of China, Qingdao, China; 2 College of Physical and Environmental Oceanography, Ocean University of China, Qingdao, China; 3 South China Sea Environmental Monitoring Center, South China Sea Branch of the State Oceanic Administration, Guangzhou, China; 4 East China Sea Environmental Monitoring Center, East China Sea Branch of the State Oceanic Administration, Shanghai, China; National Institute of Water & Atmospheric Research, New Zealand

## Abstract

Marine ecosystem is a complex nonlinear system. However, ecosystem health assessment conventionally builds on a linear superposition of changes in ecosystem components and probably fails to evaluate nonlinear interactions among various components. To better reflect the intrinsic interactions and their impacts on ecosystem health, an ecosystem coordination index, defined as the matching level of ecosystem structure/services, is proposed and incorporated into the ecosystem health index for a systematic diagnosis in the Pearl River Estuary, China. The analysis results show that the ecosystem health index over the last three decades decreased from 0.91 to 0.50, indicating deteriorating from healthy to unhealthy status. The health index is 3–16% lower than that calculated using the common method without considering ecosystem coordination. Ecosystem health degradation in the Pearl River Estuary manifested as significant decreases in structure/services and somewhat mismatching among them. Overall, the introduction of coordination in ecosystem health assessment could improve the understanding of the mechanism of marine ecosystem change and facilitate effective restoration of ecosystem health.

## Introduction

One of the global changes in ecosystem is that human activities usually have impaired ecosystem structure/services to different extents [1], especially at those estuaries where intensive exploitations were performed. The changes have raised increasing concerns and the critical question is how to holistically evaluate their impacts on ecosystem health. So far, ecosystem health assessment (EHA) at estuary focused on examining health deviation [2] from original/desired status. A relatively simple EHA is to examine the health deviation of several indicator species [3].More comprehensive EHA extends to examine the deviation of multiple ecosystem parameters including nutrient, primary productivity, biodiversity, and/or habitat [4], [5]. Even so, the EHA still might not reflect the holistic health of a whole system. Because the estuarine ecosystem is a nonlinear system, of which the structure/services are interacting in complex dynamic ways[6]–[8].When the ecosystem structure/services are damaged to different extents, consequent mismatching among them tends to result in dysfunction, even a sudden collapse of ecosystem [9].However, previous EHA is mostly based on a linear superposition of changes in the selected ecosystem parameters and probably fails to evaluate the nonlinear interactions within ecosystem.

In fact, understanding the complex relationship within an ecosystem is one of the priorities and major challenges in the earth system research today [10]. Given that ecosystem degradation often occurs as syndromes of simultaneous declines in multiple structure and services [11], a holistic evaluation of the health deviation of ecosystem structure/services from the desired status could be taken as prerequisites to quantify their interactions [1]. Further, an overall measure on the interactions among these structure/services, instead of between two or few services, is highly required. In socioeconomic researches [12], a coordination index is widely used to assess the matching level of population and economy. Referred to those studies, an ecosystem coordination index (ECI) is calculated to quantify the matching level of ecosystem structure/services and is incorporated into EHA. The new approach is an effort to obtain a holistic diagnosis of ecosystem health, which could avoid insufficient evaluations which may mislead decision-makers.

## Methodology

### 1) Study Area

**Figure 1:**
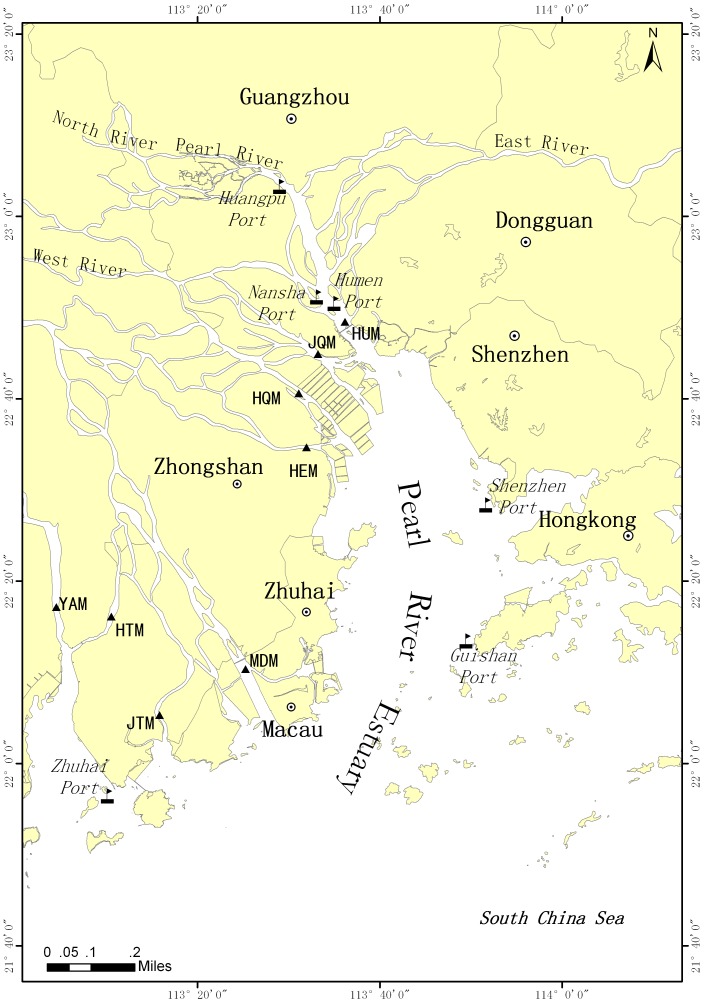
Map of Pearl River Estuary.

The Pearl River Estuary (PRE) is one of the three ecological monitoring zones in China estuary (established by China’s State Oceanic Administration in 2004). During the monitoring project at this estuary permitted and implemented by the State Oceanic Administration, environment and species protection have been well considered. The field studies did not involve endangered or protected species. No specific permissions were required for the data collection.This study is conducted in the PRE (114°37′21″–113°06′48″E, 22°49′00″–22°48′53″N), covering ∼8,000 km2 area with a coastline of about 1059 km (Fig. 1). The Pearl River is the second largest river in China in terms of fresh water discharges of about 3.3×10^11^ m^3^a^−1^. Its annual sediment flux is approximately 8.5×10^7^ tons. The Pearl River consists of three major tributaries, forming a complex river network system, which discharges into PRE from eight outlets, i.e. Yamen (YAM), Hutiaomen (HTM), Jitimen (JTM), Modaomen (MDM), Hengmen (HEM), Hongqimen (HQM), and Jiaomen (JOM). The PRE is shallow (mean depth <15 m, inner bay <5 m), with annual average surface sea temperatures (SST) ranging between 15.57 and 30.88°C, surface sea salinity (SSS) values between 21.34 and 30.62, and ratios of tidal ranges between 0.53 and 1.41. In August (wet season), the residual currents of upper and bottom estuary reach 77 cms^−1^ and 51 cms^−1^, respectively [13]. This estuary is a national nature reserve of *Sousa chinensis* (Chinese White Dolphin) that frequented by500–1000 dolphins [14]. Futian national reserves of mangrove swamp and Ao’qi provincial reserves of mangrove swamp are distributed in this estuary, which are an important habitat for creatures in South China Sea.

The PRE is suffering great pressures from the socioeconomic development of the Pearl River Delta (PRD). The PRD, accounted for 9.6% of the Gross Domestic Product (2009) in China, has become a key economic zone of electronic and automobile manufacturing, petroleum, and shipping as pillar industries over the past three decades. Meanwhile, ecological changes were drastic in this estuary, e.g. shrinking by 50% of natural mangrove wetland around Futian [13], large influx of pollutants including dissolve inorganic nitrogen, total petroleum hydrocarbon (TPH) and heavy metals etc. (786,149 tons a^−1^ in 2009) [15], frequent harmful algal blooms [16] and declining seafood quality [17]. To mitigate such degradation, a series of measures such as mangroves replantation, water cleaning, and the control on pollutant discharge had been taken. However, the PRD’s plan to urbanize 85% of area with an expected Gross Domestic Product of 72,500 billion in 2020 could further stress this estuary over the coming decades. A challenge in the PRE is to reinstate the ecosystem and ensure its’ sustainable development. To reach this goal, a scientific health assessment is needed to understand the past, current, and trends of ecosystem health, which could facilitate the effective stewardship for its mitigation and restoration.

### 2) Data Collection

Ecological monitoring data during 1980–2009 at the PRE, mainly including biodiversity, and water and sediment quality, were collected to study the state of the ecosystem health of PRE. Data used were mostly from internal research reports and the literature (Table S1 in File S1). The starting point of datasets was 1980 because of its special historic and ecological significance: 1) the ecosystem was affected by little anthropogenic disturbance and remained relatively stable before rapid development of the PRD since 1985, 2) the investigation of ecosystem at the PRE conducted in 1980–1981 was the first comparatively systematic monitoring program and the data are available.

### 3) Ecosystem Health Assessment Indicators System

**Figure 2:**
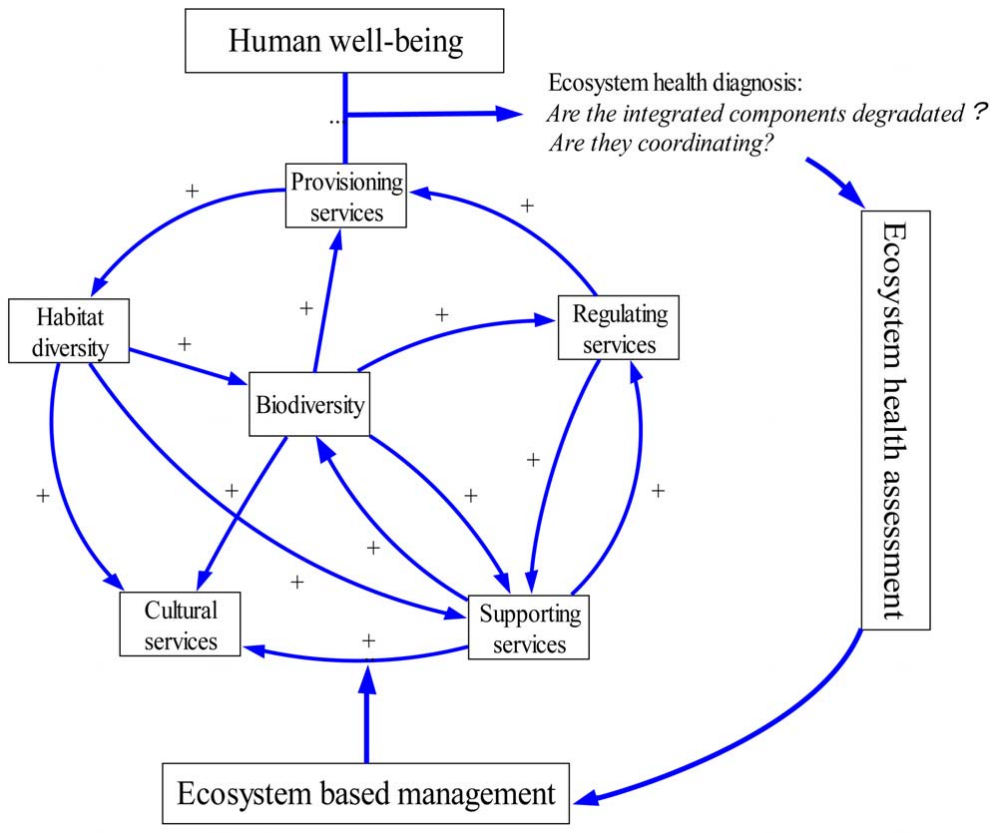
Relationship between ecosystem coordination and health assessment.

Based on the researches on interactions within marine ecosystem, we construct a conceptual model for ecosystem health assessment (Fig. 2). According to this model, a set of ecosystem health indexes (EHI) were devised and summarized in Table 1. The EHI consists of two top sections and is expressed by Formula 1. One section is defined as the ecosystem variability index (EVI), to measure the health deviation of structure and services from the desired state, including six sub-indexes based on the frame work of Millennium Ecosystem Assessment [1], i.e., Biotic structure index (BI), Habitat structure index (HI), Supporting services index (SI), Provisioning services index (PI), Regulating services index (RI), and Cultural services index (CI). The EVI is calculated using a weighted summation method (Formula 2). The weight of 30 indicators listed in table 1 is determined by a BP (back propagation) algorithm of artificial neural networks (Fig. S1 in File S1). The BP networks are a widely recommended tool in ecological models, which have advantages of dealing with information through interactions among neurons, and advantages of self-learning, self-organizing and self-adapting to uncertain system [18]. So the weight of each indicator determined by the BP algorithm tends to reflect the complex relationship among ecosystem structure/services and is relatively rational.

**Table 1:** Metrics for ecosystem health assessment in the Pearl River Estuary.

Objective (A)	Sub-objective (B)	Theme (C)	Sub-theme (D)	Indicator (E)	Ranking of indicators in layer E				unit	weight
					better	good	bad	worse		
Ecosystem Health index (EHI)	1 Ecosystem variability Index(EVI)	1.Biotic structure index (BI)	1.Species diversity	1. Phytoplankton diversity	>3	[2], [3]	[1, 2)	<1	–	0.036528
				2. Zooplankton diversity	>4	[2], [4]	[1, 2)	<1	–	0.030013
				3. Benthos diversity	>4	[2], [4]	[1, 2)	<1	–	0.033557
				4. Species of pelagic egg	≥80	(80, 60]	(60, 40]	<40	ind	0.039545
		2.Habitat structure index (HI)	2.Habitat diversity	5. Area of mangrove swamps	>450	[400, 450]	[270, 400)	<270	km^2^	0.040061
				6. Water area	>920	[828, 920)	[368, 828)	<368	km^2^	0.060285
				7.Area under marine culture	≤7000	(7000,7800]	(7800,12000]	>12000	hm^2^	0.040061
				8. Volume of freight handled in ports	≤2000	(2000,3000]	(3000,4500]	>4500	10000 tons	0.004206
		3.Supporting services index (SI)	3.Productivity	9.Primary Productivity	≥300	[270, 300)	[180, 270)	<180	mg.C/m^2^d	0.036936
			4.Water quality	10.Concentration of dissolved oxygen	≥6	[4, 6)	[3, 4)	<3	mg/l	0.055355
				11. Dissolved inorganic nitrogen	<0.20	[0.20,0.30]	(0.30,0.40]	>0.40	mg/l	0.037449
				12. Surface sea temperature	[22], [23]	(24, 25]	(24, 26]	>26	°C	0.03143
				13. Concentration of suspended substance	≤20	(20–50)	[50, 100]	<100	mg/l	0.027769
				14 Concentration of dissolved silicates	[1, 0.80]	(0.80, 0.50]	(0.50, 0.30]	<0.30	mg/l	0.026203
				15. Chemical oxygen demand	≤2	(2, 3]	(3, 4]	>4	mg/l	0.037362
			5.sediment quality	16. Total organic carbon	<1	[1], [2]	(2, 4)	≥4	%	0.008474
				17. Acid volatile sulfide	≤300	(300, 500]	(500, 600]	>600	10^−6^	0.013044
		4. Provisioning services index(PI)	6. Biomass	18. Phytoplankton biomass	≥15	[13, 15)	[9, 13)	<9	10^6^cell/m^3^	0.024226
				19. Zooplankton biomass	≥200	[180, 200)	[120, 180)	<120	mg/m^3^	0.035187
				20. Benthos biomass	≥17	[15, 17)	[10, 15)	<10	g/m^2^	0.042976
			7. Seafood quality	21. Residual level of Hg in benthic molluscs	≤0.01	[0.01, 0.05]	(0.01, 0.3]	>0.3	10^−6^ww	0.030892
				22.Residuallevel of polychlorinated biphenylsin benthic molluscs	≤5	(5, 60]	(60, 90]	>90	10^−9^ww	0.032775
				23. Residual level of Pb in benthic molluscs	≤0.1	[0.1, 2]	(2, 6]	>6	10^−6^ww	0.037894
				24. Residual level of total petroleumhydrocarbon in benthic molluscs	≤8	(8, 50]	(50, 75]	>75	10^−6^ww	0.026601
		5.Regulating services index (RI)	8. Water regulation	25. Annual runoff of Pearl River	[3300–3630]	[2900, 3300)	(2000, 2900]	<2000	billion m^3^	0.038739
				26. Rise in sea level	≤10	(10, 20]	(20, 30]	>30	mm	0.029668
			9.Disease regulation	27. Occurrence of harmful algal blooms	≤1	(1, 2]	(2, 3]	>3	times/year	0.071971
				28.Percentage of harmful algal blooms causative species	≤0.25	(0.25,1]	(1, 3]	>3	%	0.040674
		6.Cultural services index (CI)	10.Cultural heritage	29. Conserve of endangered species (Amount of aground *Sousa chinensis* )	[0, 1]	[1], [2]	[2], [3]	>3	%	0.002628
			11. Recreation	30.Concentration of total petroleum hydrocarbon in seawater	≤0.05	(0.05, 0.3]	(0.3, 0.5]	>0.5	mg/l	0.026276
	2.Ecosystem coordination index(ECI)	7. Coordination index (CoI)	12. Coordination of ecosystem structure and services	31. Dispersion coefficient of indices of BI, HI, SI, PI, RI and CI	<0.4	(0.6,0.4]	(0.9,0.6]	[1,0.9]	_	

The other top section is defined as the ecosystem coordination index (ECI) to measure the matching level among structure/services. There are many algorithms for calculating the ECI, e.g. order parameter functions, fuzzy subordinate functions and coefficients of variation (CV) [18]. The CV algorithm is good at quantifying the overall mismatching among multiple parameters and is selected to calculate the ECI of structure/services listed in section EVI (Formula 3). The ECI at 1980 is taken as health state. Lower ECI means more mismatching and lower harmony among ecosystem structure/services. Referred to the previous health assessment at estuary [19], [20], each indicator in the range of [0, 0.4], (0.4, 0.6], (0.6, 0.9] and (0.9, 1] means ill, unhealthy, sub-healthy and healthy state of ecosystem, respectively. After measuring the ecosystem health in terms of ecosystem coordination, we quantify the contribution of each structure/services to incoordination, and identify the incoordination factors influencing ecosystem health.
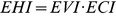
(1)

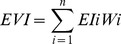
(2)

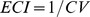
(3)where *EI* is the indexes in the EVI section, i.e. BI, HI, SI, PI, RI, and CI. *n* is the number of the indexes, i. e. 6. *CV* is the coefficients of variation among the six *EI*.

Many of these indicators were assessed by national standards, i.e. Marine Water Quality Standard (GB3097–1997), Marine Sediment Quality Standard (GB18668–2002), and Marine Biological Quality Standard (GB18421–2001). For the indicators that lack of criteria, e.g. biodiversity, biomass and cultural services, data closest to 1980 or the best state (defined as the maximum of the positive indicators or the minimum of the negative indicators) of the existing dataset were used as healthy state. The computation methods are detailed in Supporting Information.

## Results and Discussion

**Figure 3:**
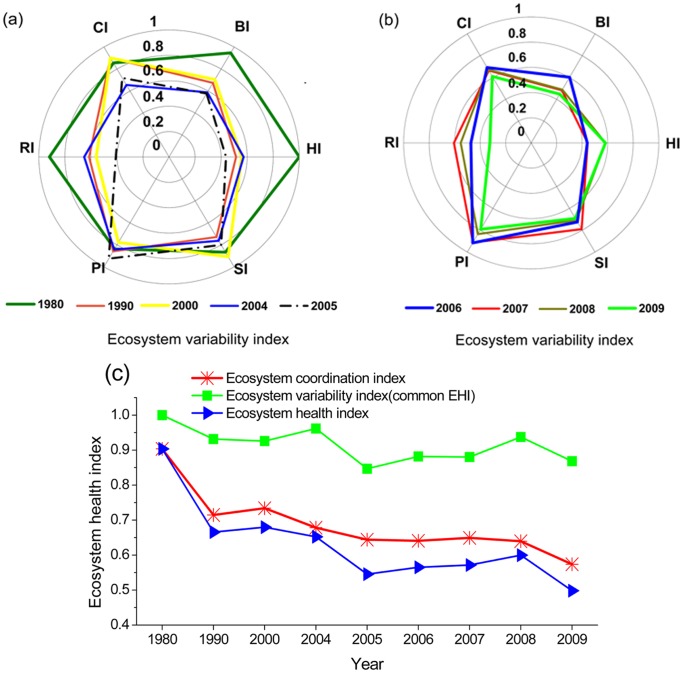
Trends of ecosystem health index in the Pearl River Estuary. (a) the ecosystem variability indexes from 1980 to 2005, (b) the ecosystem variability indexes from 2005 to 2009, (c) the ecosystem health index from 1980 to 2009.

The EHI indexes and corresponding 31 indicators at the PRE during the period of 1980–2009 are examined and shown below (Fig. 3).

### 1) Ecosystem Variability Index

a) The Biotic structure index (BI) in this study included species diversities of phytoplankton, zooplankton, and benthos. These species were selected as the key indicators because the species loss is related to the degeneration of community structure and ecosystem services [21].

The BI generally decreased from 1 to 0.44, indicating the biotic structure had been disturbed and in ill-status. Despite of a short-term recovery to healthy state (scored by 0.90) during 1998–2006, phytoplankton diversity (Shannon-Wiener) dropped to unhealthy level, e.g. 1.73 in 2009. The diversity of benthos fluctuated at sub-healthy state by 2008 but decreased to unhealthy state in 2009. The biodiversity loss could decline a bundle of ecosystem services [22], leading to instability in ecosystem health [23]. Nevertheless, remediation measures had produced certain gains in biodiversity. The zooplankton diversity started to restore from unhealthy- to sub-healthy states since 2000. After a significant decline to ill-status between 1980 and 2004, pelagic eggs caught a slight recovery in species number since 2007, suggesting an improving habitat for estuarine creatures.

b) Habitat destruction was considered among the most important stressors to biodiversity and ecosystem services. The Habitat structure index included four factors in this study, i.e., the area of mangrove swamps, water areas, areas under mariculture, and the volume of freight handled in ports.

Mangrove that destroyed in the process of reclamations was replanted. In year 2000, 2004 and the period of 2008–2009, mangrove reverted to its size in 1980, but declined almost by half in 1990 and during 2005–2007 [24]. These fluctuations might be related to the on-going reclamation, to monoculture afforestation with lower diversity [25], and mismatching among structure and services, suggesting that habitat restoration has yet to succeed. Mainly due to the reclamation [26], the water surface has been dwindled by 15% since 2000 [27] and it scored as sub-health. The habitat loss is considered a great threat to biodiversity. Narrow and shallow waters have been causing a substantial change in original physical environments, e.g., changes in SST, salinity, and nutrients [28].

As a major factor to habitat transformation in the PRE [29], the area under mariculture has increased by seven times to 4.7×10^4^ hm^2^ (1990–2006) and ranked as ill level. The increasing farms resulted in not only habitat fragmentation (e.g. breeding raft frames interrupt migration routes), but also habitat simplification (e.g. reduce wildlife habitats). However, farming areas has decreased by 14% during 2007–2009 when habitat transformation might be mitigated accordingly.

In this busy port, the volume of freight handled in ports could reflect habitat-altering for sound-sensitive organisms such as *Sousa chinensis* [30]. The port’s throughput has increased about thirty times over the past three decades and scored as unhealthy state, suggesting great changes in habitat and an increasing risk to *Sousa chinensis* etc.

Overall, the HI decreased from 1 to 0.58, indicating that the HI was approaching unhealthy level. Restoration actions having been taken did not change the decreasing trend. However, they did prevent HI from decreasing straightforward.

c) Supporting services are essential to the other services [1] and measured by productivity, water quality and sediment quality in this study.

i) Primary productivity (PP) is one of the indictors that measure the energy and substance flow in the estuary ecosystem and the annual PP changed markedly and was in an unhealthy state. This result is in accordance with previous observations, e.g. PP increased from 309 (1985) [31] to 510.8 mg.C/m^2^.d (2006–2008) [32]. The variation in PP might be associated with the changing habitat, biodiversity, and parameters of water quality, e.g. transparency, SST [33], and nutrients [34].

ii) Water quality contains six parameters, i.e., dissolved oxygen (DO), dissolved inorganic nitrogen (DIN), surface sea temperature (SST), suspended substance (SS), dissolved silicate (DSi), and chemical oxygen demand (COD) in the seawater. Most of these are commonly used in evaluating water quality, e.g. in Marine Water Quality Standard GB 3097–1997.

Concentrations and durations of exposure of DO are key factors in determining the degree of ecosystem degradation [35]. UNEP listed the PRE as “dead zones” (generally with DO below 2 mg/l) in 2006. However, we observed that hypoxia in the PRE tends to be localized, temporal and less severe. The reason is detailed as below: 1) During 2004–2009, hypoxic water (DO <2 mg/l) was seldom found, which is consistent with previous studies [36], [37]; 2) average DO exceeded 6 mg/l during springs, fail below 6 mg/l but above 4 mg/l in summer. During the summer, low oxygen waters(3–4 mg/l) occurred but reduced from 12% to 8% of our 26 sampling stations near the bottom of the estuary; 3) it is usual that the response of ecosystem to oxygen depletion is mortality of benthic organisms [35], while the diversity and biomass of benthos generally remained at sub-healthy level at the PRE during 1980–2008; 4) No significant influence on PP by hypoxia was found [38]. We proposed that the disappearance of hypoxia events might be due to the regulating services provided by the PRE. In this shallow and well-mixing estuary, the strong bottom current (25–50 cms^−1^), the short residence time (3–5d) [39], freshwaters and upwelling that intermittently disrupted stratification and aerated bottom waters [40], jointly reduced the hypoxia. Unlike PRE, the regulations at the Mississippi estuary were poor because of the weak bottom current (2–5 cm s^−1^) and the long residence time (95d), leading to frequent and severe hypoxia (DO <2 mg/l) [41], which has affected benthic communities [35]. Moreover, at the Yangtze River estuary which was considered as a “dead zone”, severe hypoxia events were resulted from stronger seasonal stratification and enhanced by the plume and Taiwan warm current [42].

It is interesting that mechanisms of hypoxia at the PRE have been changing. The PR runoffs dropped but with more nutrients and oxygen-poor waters probably expanding hypoxia areas at the PRE. As a result, most of the low oxygen areas are located in the upper reach [43] and the vicinity of HUM, JIM, HQM, HEM, or YAM outlets [13] in recent years across all seasons [43], but the hypoxic areas were mainly distributed at bottom estuary during summers of 1980s and 1990s [36].

Eutrophication is a challenge for ecosystem protection at the PRE. DIN is used to indicate eutrophication status. The mean level of DIN exceeded 0.50 mg/l (the worst grade in GB 3097–1997), referring to ill level. In the surface layer, the DIN was obviously higher than that at bottom in recent years [13], and the DIN was higher especially at nearshore and river outlets in 2004–2009, suggesting river runoffs adversely acts as a stressor to this eutrophic estuary. The direct consequences, e.g. algal blooms, eutrophication-induced hypoxia, could alter the ecosystem’s state and prevent full recovery [35].

It is worth noting that the DSi was reduced by ∼ 70% over the past three decades, mainly because that ∼ 65% of sediment fluxes carried by the PR had deposited in reservoirs since 1990s. New dams planned in the future might add to the decrease. Dominants could gradually shift from diatoms to pyrrophytas with a large decrease of Dsi [13] and lead to great changes in the community structure. Consequently, PP and biomass might be changing at the PRE since diatoms make up∼78.27% of the species and ∼99.80% of the biomass [13].

The SS and COD recovered to healthy state in 2008. The maximum interannual change in SST was 3.5°C during 1980–2009 at the PRE. This change was associated with global warming(*r* >0.75) [44] and reclamation that shallows waters [26].

iii) The total organic carbon (TOC) and acid volatile sulfide (AVS) in the sediment were used to measure the accumulated organic matters (OM) in this study. TOC recovered from sub-healthy (1990) to health state since 2004. This recovery might be due to the reduction of the two major sources of OM (52% from land and 48% from aquatic [45]). For instance, the COD from the PR had decreased by 32% and TPH had decreased by 33% during 1985–2008. The drop of phytoplankton biomass [13] contributed less to organic detritus.

The AVS maintained at healthy level, but it increased from 29×10^−6^ to 130×10^−6^ (dry weight), ranking as medium-high level among semi-closed seas in China [46]. In the case that major OM sources decreased, the AVS likely came from submarine outfalls of adjacent cities [47] or resuspension of sediment and those transported by sea current. As a combination of toxic heavy metals, the increasing AVS could pose potential risk to estuary health.

Overall, the SI ranged from 1 to 0.69, indicating a sub-healthy level.

d) Provisioning services was measured by biomass and sea food quality in this study, which can assess ecosystem changes and threats to human health [48]. Changes in biomass of phytoplankton, zooplankton and benthos were observed at the PRE. Considering that electronic, automobile and petroleum manufacturing, and shipping are main stressors from industries in the PRE, concentrations of heavy metals, polychlorinated biphenyls (PCBs), and TPH in benthic molluscas were examined. Given the higher toxic coefficient (TC) and enrichment coefficient (EC) in this estuary, the selected metals were Hg (TC = 40, EC = 4.1×10) and Pb (TC = 5, EC = 8.2×100).

The biomass of benthos and zooplankton approached their original state. However, phytoplankton experienced two decreases to ill level in 2000 and during 2008–2009, likely because of and the fluctuating factors, e.g., COD, salinity [49], DSi, and SST.

As to residual pollutants in benthic molluscas, PCBs evidently returned from unhealthy to healthy state since 2005.Hg returned from unhealthy to healthy state during 1990–2007, but fell to sub-healthy state between 2008 and 2009. TPH increased and was in sub-healthy state since 2000. Pb fluctuated between unhealthy and sub-healthy states for nearly thirty years. These results suggested a certain extent of success in controlling release of heavy metals and persistent organic pollutants to the PRE.

However, the amount of heavy metals in the PRE sediment, especially Hg [50] Pb [51] and PCBs [52] was still higher than those in the Yellow River and Yangtze Estuary. These metals could increase potential risk to seafood safety.

Overall, the PI ranged from 0.83 to 0.79, indicating as sub-healthy level.

e) Regulating services can drive significant effects on multiple services through interacting ecosystem processes [8], which mainly contain water regulation and disease regulation in this study.

i) Water regulation was analyzed as below: 1) the PR diluted water plays an important role in dealing with excessive nutriments and pollutants, maintaining suitable condition (SST, salinity [53]etc) for estuarine organisms; 2) annual PR runoffs dropped to unhealthy level, suggesting that self-purification capacity of the PRE was poor; 3) sea-level rise could cause seawater intrusion that has been a great stress to the habitat in the PRE and adjacent delta [53], and was used to reflect the estuary’s ability to regulate water-saline balance. Response to global warming, sea-level increased about 3.0 ∼ 3.6 mm a^−1^in the PRE during 1975–2006 [54]. In 2009, the relative sea-level was 88 mm higher than the average value [55], ranking as ill level. It is forecast that this rise will exceed 0.3 and 0.5 m by 2030 and 2050 [54], [56], respectively, which might alter biotic community and its distribution.

ii) For disease regulation, occurrence of HABs and percentage of HABs causative species were ranked as ill status, indicating a compromised ecosystem to diseases. The eutrophication-driven HABs were frequent and appeared to be in line with economic growth in the PRD [57]. The increase of HABs causative species might be responsible by biodiversity loss [22].

Overall, the RI ranged from 0.95 to 0.32, suggesting a vulnerable ecosystem with weak regulating functions.

f) Cultural services, usually outside ecosystem assessment, yet affecting human-being in multidimensional way, are gradually considered as the important lever for restoring other services [9]. In this case, the CI involving conservation of endangered species and recreation, were quantified by the amount of aground *Sousa chinensis* and the concentration of TPH in sea water (USEPA 2007). Aground *Sousa chinensis*increased from 1∼2 per year to about 40 in total from 2003 to 2009 [58] and scored as ill level, indicating that the lethal factors to dolphins, such as deteriorated water quality [59], confliction with ships [30], or marine engineering blasting, should still be concerned. During the past thirty years, TPH fluctuated at sub-healthy status because of continuous inputs from shipping, sediment [60] and river. These declines might reduce attractions and opportunities for public being close to water for sightseeing or swimming. The CI decreased from 0.88 to 0.60, referring to an unhealthy state.

Over the last three decades, the EVI ranged from 0.94 to 0.57, suggesting an unstable and sometimes unhealthy ecosystem. Although the habitat structure restored to some extents, supporting services and providing services maintained at relatively better level, biotic structure, regulating abilities and cultural services decreased.

### 2) Ecosystem Coordination Index

The EVI mentioned above is a linear superposition of changes in ecosystem structure/services and often used in common EHA. Yet the common assessment has overlooked the fact that ecosystem structure/services are nonlinearly interacting. The interactions can be found from the correlations between ecosystem parameters in the PRE. For example, the reduction of species of pelagic eggs strongly depended on variables of SST, TPH and areas under mariculture (*r* ^2^ = 0.86, *p*<0.05).The TOC and the occurrence of HABs determined the zooplankton diversity (*r^2^* = 0.92, *p*<0.01). In addition, the phytoplankton diversity varied with SST(*r* = −0.70, *p*<0.05)and DSi(*r* = 0.70, *p*<0.05). Also, the diversity of zooplankton and phytoplankton, the COD and the area of mangrove swamps were found to be determinants of frequent HABs (*r* ^2^ = 0.95, *p*<0.01), and the increase of causative species of HABs was associated with the TPH (*r* = 0.60, *p*<0.05).Additionally, it has been reported that changes in COD and salinity could decrease the biomass of phytoplankton and benthos [49] in the PRE. TPH [61], PCBs [62], Hg, and Pb [59] enriched in dolphins lead to a high mortality of newborn dolphin.

These correlations revealed that the complex interactions within ecosystem could also be associated with health degradation. It appears that one decreasing structure/services can impair multiple structure/services. For instance, the deteriorating water (caused by the increasing COD) lead to frequent HABs and biodiversity loss. It can also be speculated that a decrease in multiple structure/services can synergize the drop of a kind of structure/services, e.g., the habitat destruction (caused by increasing areas under mariculture), and the worse water quality (caused by increasing SST and TPH) can together decrease the species of pelagic eggs. Moreover, the interactions among ecosystem structure/services could be bidirectional [8]. For example, the weak disease regulation (indicated by the occurrence of HABs) might decrease the zooplankton diversity. In turn, the loss of zooplankton diversity, implying a decline phagocytosis to harmful species, could further weaken disease regulation. Such positive feedback loops generally exist in the interdependent structure/services, e.g. habitat/species diversity, and supporting services, which is tightly related to health changes. These nonlinear dynamic and feedbacks suggest that ecosystem degradation is initiated by environmental perturbations, and automatically enhanced by the mismatching among ecosystem structure/services. Ecosystem degradation could occur as a decrease in structure/services, or as a mismatching among them. To what extent they match was expressed by the ECI proposed in this study, which could be a prediction of health trends from interior profile.

**Figure 4:**
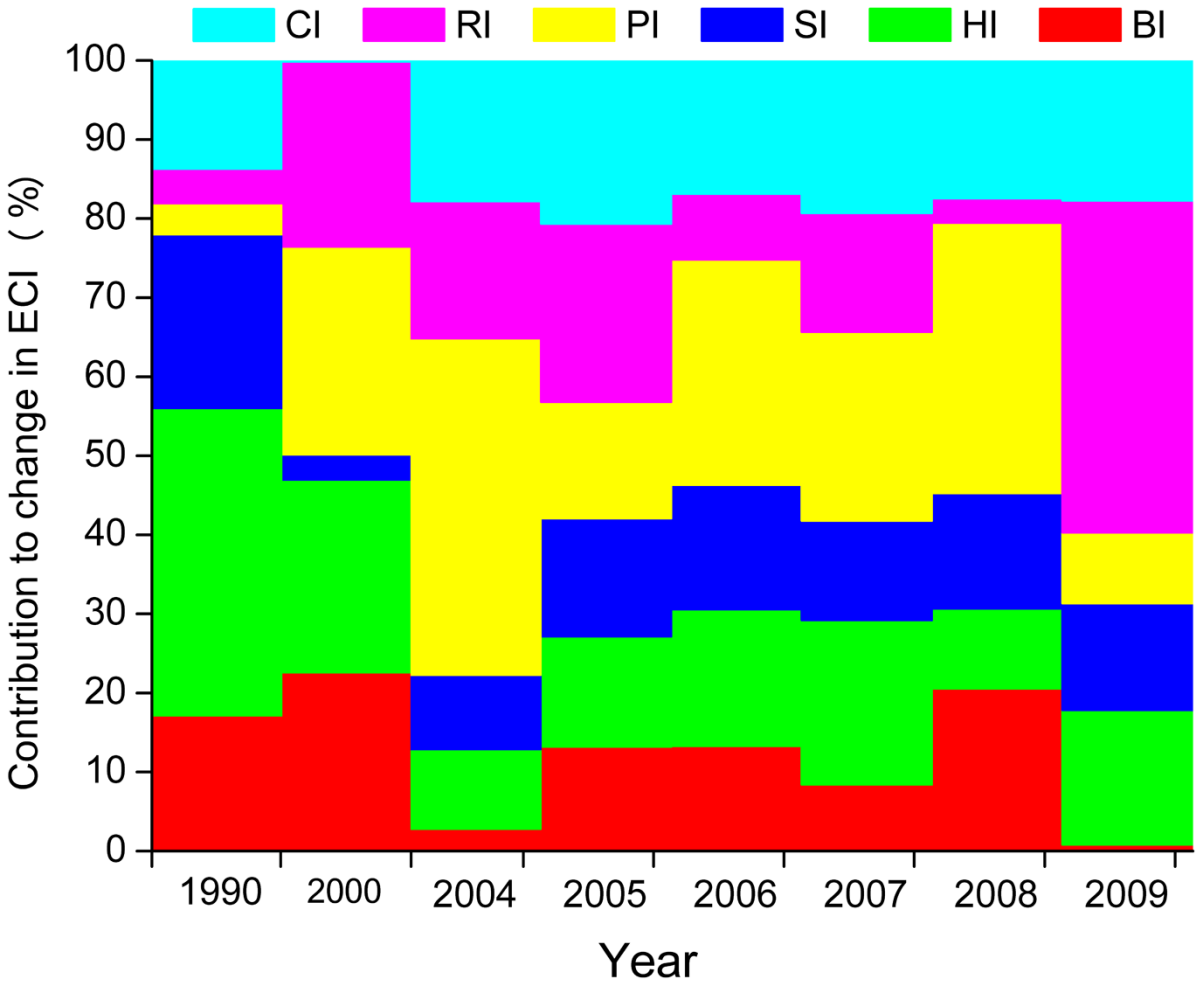
Contributions of ecosystem structure and services to coordination.

During the period of 1980–2009, the ECI ranging from 1 to 0.86 showed a certain extent of discordance of ecosystem. To obtain the contribution of each structure/services to mismatching, we compared the ecosystem coordination including and excluding a certain structure/services, respectively. Approaches are detailed in the supporting information. The results (Fig. 4) showed that the contribution of habitat structure (in 1990), providing services (in 2000, 2004, 2006–2008), and regulating services (in 2009) could reach 38%, 42%, and 42%, respectively, which in sequence acted as the most uncoordinated factor during 1980–2009. During these decades, the biodiversity accounted for 1–22% of incoordination, and the supporting services’ contributions to mismatching ranged from 3 to 13%, which were relatively minor contributors. Cultural services’ contributions were steady at about 17%. The contribution of providing services to mismatching markedly reduced from 42% to 8%, and that of habitat structure reduced from 38 to 17%. While the regulating services that contributed 42% to incoordination were the most uncoordinated factor at present. To obtain the overall harmony of ecosystem, the PRE should give priority to restoration of regulating ability.

On the whole, the EHI ranging from 0.91 to 0.50 was scored, suggesting an unhealthy state (Fig. 3c). Healthy status of ecosystem at the PRE manifested as a significant decrease in structure/services and somewhat mismatching among them. Compared to the common EHI based on linear superposition of changes in ecosystem parameters (i.e. just considering EVI), the EHI calculated by our approach in view of ecosystem coordination is 3–16% lower. The lower EHI in this study might provide a new perspective to understand the ecosystem degradation caused by internal interactions.

### Summary

In this study, ecosystem health assessment is extended to include the nonlinear interactions within the ecosystem. By incorporating the ecosystem coordination index into a health diagnosis of ecosystem structure/services, we attempted to assess ecosystem health from external and internal perspectives, and to illustrate the past, state, and trends of ecosystem health and their causes systematically in the PRE. Moreover, by comparing contributions of each factor to ecosystem incoordination, our study could identify the most important uncoordinated factor influencing ecosystem health, thereby provide a suitable option to prioritize restoration and sustainably managing ecosystem.

Particularly, being affected by anthropogenic perturbations that superposed on natural changes, the ecosystem in the PRE was found swinging from healthy to unhealthy state during the past three decades. Ecosystem structure/services decreased to various extents, i.e. ranging from sub- to un-healthy states. Additionally, the mismatching level of structure/services was at sub-healthy level, which is also an important sign of ecosystem degradation which should not be neglected.

Overall, assessing ecosystem health focused on interactions among structure/services might raise some urgent research questions for future EHA. For instance, how different ecosystem structure/services dynamically coordinate with others at different scales (spatial and time); and to what extent they are affecting ecosystem health.

## Supporting Information

File S1 **Table S1. Data collection and sources.** Data sets on biodiversity, water quality and sediment quality were derived from ecological surveys during 1980–2009 at Pearl River Estuary. **Figure S1. A conceptual model of BP algorithm.** A standard feed-forward back-propagation artificial neural network consists three layers, i.e. an input layer, a hidden layer and an output layer, by which the weights of assessment indicators were determined.
(DOC)
