# Supplementary material for: Ecosystem Health Assessment in the Pearl River Estuary of China by Considering Ecosystem Coordination
Source: PLoS One. 2013 Jul 23;8(7):e70547. doi: 10.1371/journal.pone.0070547 (PMC3720912; doi:10.1371/journal.pone.0070547)
Supplement: File S1 — Table S1. Data collection and sources. Data sets on biodiversity, water quality and sediment quality were derived from ecological surveys during 1980–2009 at Pearl River Estuary. Figure S1. A conceptual model of BP algorithm. A standard feed-forward back-propagation artificial neural network consists three layers, i.e. an input layer, a hidden layer and an output layer, by which the weights of assessment indicators were determined. (DOC) [file pone.0070547.s001.doc]

**File S1: Supporting Information**

Ecosystem health assessment in the Pearl River Estuary of China by considering ecosystem coordination

Xiaoyan Chen^1^, Huiwang Gao^1*^, Xiaohong Yao^1^, Zhenhua Chen^2^,

Hongda Fang^3^ , Shufeng Ye^4^

1. Key Laboratory of Marine Environment and Ecology (Ocean University of China)

Ministry of Education of China, Qingdao, China

2. College of Physical and Environmental Oceanography

Ocean University of China, Qingdao, China

3. South China Sea Environmental Monitoring Center

South China Sea Branch of the State Oceanic Administration, Guangzhou, China

4. East China Sea Environmental Monitoring Center

East China Sea Branch of the State Oceanic Administration, Shanghai, China

Number of pages: 8

Number of tables: 1

Number of figures: 1

1. **Data collection and sources**

**Table S1.** Data collection and sources

| **Year** | **Times of survey cruises** | **Number of survey stations** | **Data sources** |
| --- | --- | --- | --- |
| 1980-1985 | 12 (monthly)  4 (seasonal) | 28-34 (biological survey)  22 (water quality)  15 (sediment quality) | *(1)* |
| 1990-1991 | 4 (Summer, Winter) | 18-23 (water quality)  15-25 (biological survey)  25 (sediment quality) | *(2)* |
| 2000-2001 | 2 (April, August) | 26 (water quality)  26 (biological survey)  8-26 (sediment quality) | *(3)* |
| 2004-2009 | 10 (April, August) | 12 (water quality)  27 (biological survey)  13 (sediment quality) | *(4)* |

**2. Calculation methods for each factor**

**2.1 Calculation methods for indicators in layer E (Table 1)**

Adopting threshold method in dimensionless treatment, the initial data were computed by using formula S (1ab).   

where *x_i_’* is the standardized value of *i^th^* indicator, *x_i_* is initial value of *i^th^* indicator, x*_min_* is the minimum of *i^th^* indicator, and x*_max_* is the maximum of *i^th^* indicator. Formula S(1*a*) is designed for positive indicators (positive ecological effects occur when the indicator values increase, e.g., dissolved oxygen); formula S(1*b*) is designed for the negative indicator (negative ecological effects occur when the indicator values increase, e.g., chemical oxygen demand).

 **2.2 Calculation methods for ecosystem variability index**

Ecosystem variability index (EVI) and the six sub-indexes, i.e., Biotic structure index (BI), Habitat structure index (HI), Supporting services index (SI), Provisioning services index (PI), Regulating services index (RI), and Cultural services index (CI) are calculated using formula S(2), where *W_i_* is the priority of *i^th^* indicator, obtained by BP algorithm of artificial neural networks, *n* is the indicator number of *EI*.

A score of 1 is assigned to *EI* of 1980, the desired end point. Based on the deviation from the restoration targets, *EI* computes a number between 0 (worst) and 1 (best) and is assumed to be failure, caution or success when it increases from 0, to 0.4, 0.6 and 0.9. Each index included in Table 1 in the range of [0-0.4], (0.4, 0.6], (0.6, 0.9] and (0.9, 1] means ill, unhealthy, sub-healthy and healthy state of ecosystem, respectively.

The weights of 30 indicators listed in Table 1 were determined by a standard feed-forward back-propagation artificial neural network (BP ANN) (Figure S1). The ANN consists three layers: an input layer, a hidden layer and an output layer. Its input layer consists of source nodes, and its output layer consists of neurons; these two layers connect the network to the outside world. In addition to these two layers, the BP ANN usually has one or more layers of neurons referred to as hidden neurons because they are not directly accessible. The hidden neurons extract important features contained in the input data *(5)*.

o

input layer

hidden layer

output layer

Figure S1. A conceptual model of BP [algorithm](app:ds:algorithm)

The multilayer perceptron is usually trained by a back-propagation algorithm with feed-forward and backward phases. First, one estimation subset of the examples is used for model training, and one validation subset is used for evaluating model performance. The neural network is optimized using a training data set. A separate test data set is used to halt training to mitigate over-fitting. The training cycle is repeated until the test error no longer decreases *(5)*.

(1)Feed-forward phases

*X*(*x_1_,x_2_,……x_n_*) are input vectors, *Y*(*y_1_,y_2_,…y_m_*) are hidden vectors, *o* is output vector. For the output layer,

For the hidden layer,

For formula S (3) and S (5), a sigmoid function S (7) and its derived function S (8) are adopted as the activation function of various neurons in BP network.

$$\begin{matrix} f(x)=\frac{1}{1+e^{-x}} (7) \\ f'(x)=f(x)[1-f(x)] (8) \end{matrix}$$

(2)Back-propagation phases

This process is referred to as “reverse spread of error”. The error is the difference between the actual output and the expected output. According to the least mean-square error principle, the error could be reduced by modifying the linking weight from the output level to the intermediate level. Errors of output layer are calculated by formula S (9), that of hidden layer by formula S (10), and that of input layer by formula S (11).

As showed by Formulas S (9-11), the network error is a function of weights *w_jk_* and *v_ij_* of various layers. To reduce the errors, a gradient method is adopted to modify the network linking weight in BP learning algorithm (formula S12). Where constant$\eta\in(0,1)$is the learning rate.

For output layer and hidden layer,

The calculation formulas for modifying the network errors are as below.

An error signal is defined for output layer and hidden layer, respectively, by formula (S13):

Then formula (S12) could be revised as formula (S14):

The modification of weight could be calculated form error signal $\delta_{k}^{o}$ and$\delta_{j}^{y}$. For output layer and hidden layer, the two error signal could be calculated by formula S15:

According to formula S (9-10), as for output layer and hidden layer, obtain,

According to formula S (15) and S (8), obtain,

According to formula S (13), S (14), and S (17), the weight modification formulas are:

Then the modified weights are obtained by formula S (19), where *p* is the number of learning samples.

Overall, the weight of indicators in the input layer could be obtained by formula S(20):

In this study, a BP ANN was constructed with an input layer consists of 30 source nodes (i.e.30 indictors), a hidden layer consists of 23 nodes (75% of source nodes, set by experience) *(6)*, and an output layer consists of 1 neurons (i.e. *EVI*). For indicator in every grade (Table 1), 30 learning samples were random generated. There are 4 grades in this assessment, i.e. health, sub-health, ill-health, and ill, so 120 samples were generated in total. For each input sample, an expected output value was set based on the corresponding *EVI* score, e.g. for the input indicators in sub-health grade, which expect value was in the range of [0.6, 0.9). Initial weights of indicators were random generated from 0.1 to 0.5. The learning rate$\eta$was set as 0.9. The allowable error was 0.0001, and the maximum iterations were 1000*(6)*. By this BP algorithm, weights of 30 indictors were obtained and listed in Table 1.

**2.3 Calculation methods for ecosystem coordination index**

Ecosystem coordination index (ECI) is measured by coefficient of variation (formula S21-24). Where *ECI* is ecosystem coordination index, σ is standard deviation, $\overline{x}$ is average value. The higher of *ECI* means less divergence and better harmony among ecosystem elements.

$$ECI=1/CV (21)$$

$$CV=\frac{\sigma}{\overline{x}}\times100\% \left(22\right)$$

$$\overline{x}=\frac{1}{N}\sum_{i=1}^{N} x_{i} (23)$$

$$\sigma=\sqrt{\frac{\sum_{i=1}^{n} (x_{i}-\overline{x})^{2}}{N}} (24)$$

**2.4 Calculation methods for ecosystem health index**

Ecosystem health index is measured by formula S25. Where *EHI* is ecosystem health index, *EVI* is ecosystem variability index, *ECI* ecosystem coordination index.

$$EHI=EVI\cdot ECI (25)$$

**3. Contributions of ecosystem components to** **mismatching**

To under how dysfunction affecting ecosystem services, we compared coordination of ecosystem in- and excluding the structure/services respectively using formula S26, and obtained their contributions to mismatching.

Where *C_i_* is **c**ontributions of *i^th^* ecosystem component (i.e. biotic structure, habitat structure, supporting services, provisioning services, regulating services, or cultural services, respectively) to mismatching, is coordination of ecosystem including all components. is coordination of ecosystem excluding *i^th^* component.

**References:**

(1) LGCIPRE (leading group of comprehensive investigation on the coastal and tidal flat resources in the Pearl River Estuary of Guangdong Province) (1985) Report of comprehensive investigation on coastal and tidal flat resources in the Pearl River Estuary. Guangzhou: Guangdong science and technology press.

(2) LGCIPRE (leading group of comprehensive investigation on the coastal and tidal flat resources in the Pearl River Estuary of Guangdong Province) (1995) Report of comprehensive investigation on island resources in the Pearl River Estuary. Guangzhou: Guangdong science and technology press.

(3) SCSEMC (2000) The study on improvement of marine environmental monitoring system for the Pearl River Estuary in the People Republic of China. Guangzhou: South China Sea Environmental Monitoring Center.

(4) SCSEMC (2009) Reports of the Prearl River Estuary Ecological Monitoring Zone. Guangzhou: South China Sea Environmental Monitoring Center.

(5) Shi H, Lee K, Lee H, Ho W, Sun D, Wang J and Chiu C. (2012) Comparison of Artificial Neural Network and Logistic Regression Models for Predicting In-Hospital Mortality after Primary Liver Cancer Surgery. *PLoS ONE*, 7(4): e35781.

(6) Tang QYAF (2007) DPS Data processing system: Experimental design, statistical analysis, and data mining. Beijing: Science Press.
